# Molecular Evolution of Multiple Arylalkylamine *N*-Acetyltransferase (AANAT) in Fish

**DOI:** 10.3390/md9050906

**Published:** 2011-05-24

**Authors:** Bina Zilberman-Peled, Sharron Bransburg-Zabary, David C. Klein, Yoav Gothilf

**Affiliations:** 1 Department of Neurobiology, George S. Wise Faculty of Life Sciences, Tel Aviv University, Tel Aviv, 69978, Israel; E-Mail: yoavg@tauex.tau.ac.il; 2 Bioinformatics Unit, George S. Wise Faculty of Life Sciences, Tel Aviv University, Tel Aviv, 69978, Israel; E-Mail: sharron.zabary@gmail.com; 3 Section on Neuroendocrinology, Office of the Scientific Directory, National Institute of Child Health and Human Development, National Institutes of Health, Bethesda, MD 20892, USA; E-Mail: kleind@mail.nih.gov

**Keywords:** seabream, serotonin, dopamine, gene duplication

## Abstract

Arylalkylamine *N*-acetyltransferase (AANAT) catalyzes the transfer of an acetyl group from acetyl coenzyme A (AcCoA) to arylalkylamines, including indolethylamines and phenylethylamines. Multiple *aanats* are present in teleost fish as a result of whole genome and gene duplications. Fish *aanat1a* and *aanat2* paralogs display different patterns of tissue expression and encode proteins with different substrate preference: AANAT1a is expressed in the retina, and acetylates both indolethylamines and phenylethylamines; while AANAT2 is expressed in the pineal gland, and preferentially acetylates indolethylamines. The two enzymes are therefore thought to serve different roles. Here, the molecular changes that led to their specialization were studied by investigating the structure-function relationships of AANATs in the gilthead seabream (sb, *Sperus aurata*). Acetylation activity of reciprocal mutated enzymes pointed to specific residues that contribute to substrate specificity of the enzymes. Inhibition tests followed by complementary analyses of the predicted three-dimensional models of the enzymes, suggested that both phenylethylamines and indolethylamines bind to the catalytic pocket of both enzymes. These results suggest that substrate selectivity of AANAT1a and AANAT2 is determined by the positioning of the substrate within the catalytic pocket, and its accessibility to catalysis. This illustrates the evolutionary process by which enzymes encoded by duplicated genes acquire different activities and play different biological roles.

## 1. Introduction

Arylalkylamine *N*-acetyltransferase (AANAT) belongs to the large GNAT acetyltransferases superfamily, members of which are found in all kingdoms of life [1], reflecting the fundamental importance of acetyl transfer. Members of the GNAT superfamily acetylate a wide range of substrates, ranging from small molecules to large proteins [2]. AANATs selectively acetylate arylalkylamines, including indolethylamines and phenylethylamines [3–7].

Members of the AANAT family are expressed in the vertebrate pineal gland and retina; homologs are found in amphioxus, fungi, algae and bacteria [8,9]. In the vertebrate pineal the gland, AANAT functions in the melatonin synthesis pathway by catalyzing acetylation of serotonin, resulting in *N*-acetylserotonin; the latter is *O*-methylated by hydroxyindole-*O*-methyltransferase (HIOMT) to form the hormone melatonin. Melatonin is produced in a rhythmic manner with high levels during the night and low levels during the day. These temporal changes are controlled by the activity of AANAT [10,11]. Accordingly, AANAT is considered the key enzyme in the generation of the melatonin rhythm.

AANAT is a globular protein (20–25 kDa) comprised of a catalytic domain flanked by regulatory domains. The catalytic domain includes the AcCoA binding region found in other members of the GNAT superfamily and three AANAT-defining loops that form the arylalkylamine binding pocket. The kinetic mechanism of AANAT obeys an ordered BiBi ternary complex (sequential) where the binding of AcCoA precedes the binding of the amine substrate [12]. Crystallographic analysis of the sheep AANAT indicated that the reactive groups (*i.e*., the primary amine and the acetyl group) are positioned in close proximity within the catalytic funnel [13–17]. Catalysis is initiated by deprotonation of the protonated amine, indirectly facilitated by neighboring histidines (His^120,122^). The two histidines are part of a proton wire that channels protons to the surface of the enzyme. This deprotonation leads to nucleophilic attack on the thioester bond of AcCoA. Protonation of the CoA thiolate-leaving group is facilitated by a conserved tyrosine (Tyr^168^).

In mammals and chicken, only a single *aanat* gene has been identified, which is expressed in the pineal gland and in the retina. In teleost fish, multiple *aanat* genes, *aanat1a, aanat1b* and *aanat2,* have been identified and characterized [8,18–23]. The existence of multiple *aanats* in fish is likely to be the result of a genome and gene duplications that occurred after the divergence of teleosts and the tetrapod lineage [8,24,25]. Genome duplications are important molecular evolutionary forces that allow the duplicate genes to acquire new or related functions, or to alter their expression pattern, a process known also as co-option [24,26]. Such an evolutionary process is apparent from studies on *aanat1a* and *aanat2*. These genes display different patterns of tissue expression and encode proteins that have different kinetic characteristics and substrate preference, consistent with different functions. AANAT1a is expressed mainly in the retina and to some extent also in the brain, and, like the mammalian and avian AANATs, acetylates both indolethylamines and phenylethylamines. AANAT2 is primarily expressed in the pineal gland and preferentially acetylates indolethylamines, especially serotonin [23,27]. The activity of AANAT2 is characterized by weaker affinity and higher velocity for its substrates as compared to those of AANAT1a [18,22,28]. The biological significance of multiple AANATs in fish is likely to represent evolutionary responses to different selective pressures. While AANAT2 may have acquired a higher selectivity for serotonin in order to enhance the efficiency of melatonin production [8,29], AANAT1a preserved a broad arylalkylamine acetylation capability so that it could serve multiple functions in the retina. Thus, retinal AANAT may play other roles in addition to being involved in melatonin synthesis. Through the acetylation of dopamine, retinal AANAT may contribute to the control of dopamine levels or initiate the synthesis of a novel bioactive molecule [29]. Retinal AANAT may also play a more general role in detoxification through its action on a broad range of arylalkylamines [8,30]. AANAT1b and AANAT1a are closely-related proteins with high homology and identical substrate binding and catalytic sites. Although the biochemical properties of AANAT1b are still unknown, the presence of the three AANATs in some fish species supports the hypothesis that these enzymes play multiple roles [31,32].

The presence of multiple AANATs in fish provides an opportunity to study the effect of natural mutations in these closely-related molecules. Here we investigate the structure-function relationship of seabream AANAT1a and AANAT2, by attempting to identify the molecular basis of their kinetic differences and substrate preference. Inhibition experiments were performed to assess the binding capabilities of AANAT1a and AANAT2 to their different substrates. Site-directed mutagenesis was used to determine the importance of residues in the arylalkylamine binding domain that differ between the two enzymes. In addition, the predicted structures of wild type AANAT1a and AANAT2 were compared in regard to their capacity to bind arylalkylamines. The results of these studies provide a foundation for a molecular explanation of the functional differences between AANAT1a and AANAT2.

## 2. Results and Discussion

### 2.1. Kinetic Characterization of Mutated AANAT1a and AANAT2

Fish have become important models for the evolutionary significances of gene duplication since many gene families possess more members in fish than in mammals. Two evolutionary processes can lead to a protein with a novel function: a change in the coding sequence of the gene that alters the biochemical properties of the protein, and a change in its regulatory sequence that alters its temporal or spatial expression pattern [26]. The duplicate AANATs in fish illustrate these evolutionary processes by representing differences, both in their spatio-temporal expression patterns [18,27,33,34] and in their biochemical properties [18,22,28]. The different qualities of each enzyme are thought to serve two melatonin generating systems, one in the retina and the other in the pineal gland [18,21,22]. It has also been suggested that AANAT1a is involved in dopamine metabolism and amine detoxification in the retina [8,29].

In the current study the molecular-structural base for the differences between the two enzymes was biochemically investigated, followed by *in silico* experiments.

**Figure 1.**
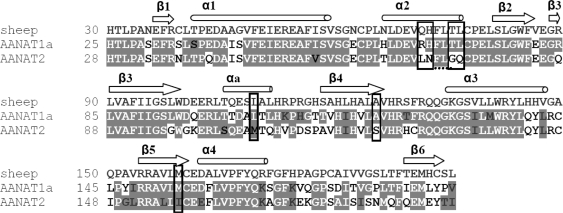
Sequence alignment of sheep AANAT, seabream AANAT1a and seabream AANAT2. The alignment starts in residue 30 of the sheep AANAT, in residue 25 of the seabream AANAT1a, and in residue 28 of the seabream AANAT2. The coloring of the alignment follows the sequence similarity between AANAT1a or AANAT2 and the sequence of the sheep AANAT; identical residues are shaded and colored in white, similar residues are shaded. The secondary structure elements, as analyzed by Hickman *et al.* [15] and Dyda *et al.* [2], are shown above the residues. The mutation sites are boxed. The pairwise alignment was done using the Needle alignment tool.

The effect of reciprocal mutation (See ‘Experimental Section’ for details, Figure 1, Table 1) on the activity of the two enzymes was measured in order to study the molecular basis that underlies the biochemical differences.

**Figure 2.**
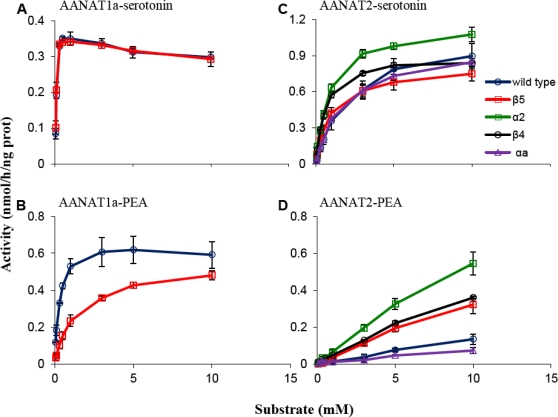
Acetylation of arylalkylamines by wild type and mutant AANATs. Activities were measured in the presence of near saturating levels of AcCoA and increasing concentration of amine substrates. The graphs represent acetylation activities (mean ± SE) with serotonin and phenylethylamine. A generally similar kinetics was found with tryptamine and dopamine. The AANAT1a- α2, β4 and αa mutants did not express well and were inactive, consistent with misfolding, and therefore were not analyzed (see ‘Experimental Section’ for details). **(A)** AANAT1a and mutants with serotonin; **(B)** AANAT1a and mutants with phenylethylamine; **(C)** AANAT2 and mutants with serotonin; **(D)** AANAT2 and mutants with phenylethylamine; PEA = phenylethylamine.

Analysis of the acetylation activity of wild type and mutant proteins was done with serotonin and tryptamine (indolethylamines) and dopamine and phenylethylamine (phenylethylamines). Figure 2 represents acetylation activity with serotonin and phenylethylamine, which are generally similar to the kinetics found with tryptamine and dopamine, respectively. Table 2 represents the catalytic efficiency in terms of Kcat/Km values. Acetylation of phenylethylamine and dopamine by AANAT2 mutants did not obey the Michaelis-Menten equation (Figure 2), and their activities are therefore expressed relative to wild type AANAT2 activity in the presence of 10 mM substrate (shaded values, Table 2); these values cannot be compared to other enzyme-substrate Kcat/Km values. The specific effect of each mutation is detailed below.

#### 2.1.1. β5 Mutations

Met^159^ is one of six residues that form the amine substrate binding pocket in sheep AANAT [15]. It forms a hydrogen bond with the nitrogen of the amine substrate and is therefore crucial for its stabilization.

The results showed that substitution of Ile to Met in AANAT2 (mutation β5 of AANAT2) increased the ability of AANAT2 to acetylate phenylethylamines by ∼2.5-fold (p < 0.05) as compared to wild type, and also increased its affinity to indolethylamines (Km = 0.90 mM *vs.* 2.0 mM of wild type for tryptamine and Km = 0.94 mM *vs.* 2.2 mM of wild type for serotonin), resulting in a 2- to 3-fold increase (p < 0.05) in the Kcat/Km values (Table 2, Figure 2). The reciprocal β5 mutation of AANAT1a (M154I) reduced AANAT1a capacity to acetylate dopamine and phenylethylamine by 3.9- and 6.5-fold (p < 0.01), respectively, but reduced tryptamine acetylation by only 1.6-fold (p < 0.05), while having almost no effect on its capacity to acetylate serotonin (Table 2, Figure 2), indicating that the decrease in phenylethylamines acetylation is specifically related to the mutation. These results suggest that the Met/Ile difference in this position contributes to the different kinetic characteristics and the low ability of AANAT2 to acetylate phenylethylamines.

The importance of the hydrogen bond formed at this site (Met^159^ in sheep AANAT) to the stabilization of the amine substrate was previously emphasized [2,15]. The contribution of this site to the catalytic mechanism of AANATs is now expanded to include determination of substrate selectivity. It is notable that an evolutionary substitution of two neutral residues, Met^154^ in AANAT1a by Ile^157^ in AANAT2, has a profound effect on substrate preference.

#### 2.1.2. α2 Mutation

The α2 region is a component of loop 1 which was recognized as the most floppy part of the enzyme [14,15,35]. The binding of AcCoA displaces loop 1 and leads to reorganization of helices α1 and α2 at both ends of the loop. This conformational change contributes to the formation of the arylalkylamine binding pocket [14,15]. Interestingly, this small region contains changes in five adjacent amino acids that characterize fish AANAT2. The mutations of α2 region changed two pairs of adjacent amino acids.

This mutation increased AANAT2 ability to acetylate dopamine and phenylethylamine by 3.80- and 4.33- fold, (p < 0.05) respectively as compared to wild type; and increased its affinity to indolethylamines (1.2 mM for tryptamine and 0.88mM for serotonin), as reflected in a 2- to 2.5- fold increase (p < 0.05) in the Kcat/Km values (Table 2, Figure 2). The α2 mutation conferred upon AANAT2 the highest increase of phenylethylamines acetylation among the four AANAT2 mutants.

The results strengthen previous studies showing the importance of this region for the catalytic action of the AANAT enzymes. Furthermore, it suggests that evolutionary changes within the α2 region of AANAT2 underlie the structural changes that may contribute to its low capacity to acetylate phenylethylamines.

#### 2.1.3. β4 Mutation

The β4 region is located within the most conserved motif of the GNAT superfamily which is the site of AcCoA binding and contains residues that participate in the catalytic reaction [2].

The results show that substitution of Ser to Ala in AANAT2 (Mutation β4 of AANAT2) increased the ability of AANAT2 to acetylate dopamine and phenylethylamine by 2.48- (p < 0.05) and 3.03- fold, (p = 0.079), respectively. This mutation also increased the affinity of AANAT2 for indolethylamines (0.65 mM for tryptamine and 0.65mM for serotonin), as reflected in the ∼3-fold (p < 0.05) increase in the Kcat/Km values (Table 2, Figure 2).

These results support previous studies showing that the β4 region is important for the catalytic reaction of the AANAT enzymes, and specifically suggest that Ala^120^ (in AANAT1a) and Ser^123^ (in AANAT2) are involved in determining the kinetics and substrate preference of these enzymes.

#### 2.1.4. αa Mutation

αa is a subdomain of the two-turn helix that is located between the β3 and β4 sheets [14]. This helix defines a “third wall” in the amine binding site that almost entirely encloses the catalytic funnel, and exists only in the AANAT family and not in other members of the GNAT superfamily [2]. Specifically, residue Leu-109 of the Sheep AANAT was reported to be part of the serotonin binding site [15]. The corresponding substitution of Met to Leu in AANAT2 (mutation αa of AANAT2) did not effect phenylethylamines or indolethylamines acetylation activities in comparison to wild type (Table 2, Figure 2). These results suggest that Leu^104^ (AANAT1a) and Met^107^ (AANAT2), which participate in the serotonin binding site, do not contribute to the differences in the substrate preference of these enzymes.

### 2.2. Inhibition Experiments

**Figure 3.**
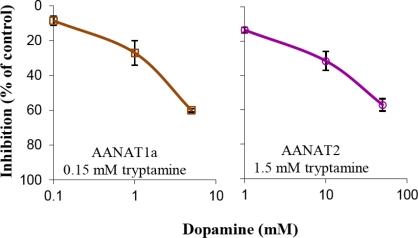
Dopamine inhibits tryptamine acetylation by AANAT1a and AANAT2. Dopamine inhibition of tryptamine acetylation was analyzed using the radiochemical assay. The concentrations of tryptamine selected approximated the Km values of each enzyme (0.15 and 1.5 mM for AANAT1a and AANAT2, respectively), in the presence of increasing concentrations of dopamine (0, 0.1, 1, 5 mM or 0, 1, 10, 50 mM for AANAT1a and AANAT2, respectively). Other conditions were as previously determined for wild type AANAT1a and AANAT2 [22]. Values are given as mean ± SE.

Two reasons may account for the low ability of AANAT2 to acetylate phenylethylamines: (1) phenylethylamines do not bind to the catalytic pocket of AANAT2; or, (2) phenylethylamines do bind to the catalytic pocket of AANAT2 but are not acetylated by the enzyme. In the latter case, an inhibition of indolethylamines acetylation would be expected in the presence of phenylethylamines. To discriminate between the two options, acetylation of subsaturating tryptamine concentrations, at about the Km value of each enzyme (0.15 mM for AANAT1a and 1.5 mM for AANAT2) was measured in the absence or presence of increasing dopamine concentrations: 0.1 mM or 1mM for AANAT1a and AANAT2, respectively (∼1×); 1 mM or 10 mM for AANAT1a and AANAT2, respectively (∼6×); and 5 mM or 50 mM for AANAT1a and AANAT2, respectively (∼30×) the tryptamine concentrations (Figure 3). Similar inhibition patterns (Pearson correlation, R = 0.87, p = 0.024) were found for both enzymes, with minimal inhibition at the 1:1 dopamine:tryptamine ratio, ∼30% inhibition at the 6:1 dopamine:tryptamine ratio, and ∼ 60% inhibition at the 30:1 ratio (Figure 3). These results suggest that dopamine binds to the catalytic pockets of AANAT1a and AANAT2 at similar affinities, which are lower than those of tryptamine.

The amine binding pocket of AANATs constitutes a small edge of the catalytic funnel that is surrounded by hydrophobic residues. The dominant factor that determines which molecules will enter the funnel is size; and the main forces that hold the amines are van der Waals’ interactions and hydrophobic packing [14,15].

The catalytic mechanism of AANAT, like other members of the GNAT superfamily, involves an acetyl transfer from AcCoA to the acceptor through a direct nucleophilic attack. Although members of the GNAT superfamily acetylate a wide variety of substrates and exhibit low amino acids sequence identity, the structural conservation of the AcCoA binding site is remarkably high [2,36]. The catalytic mechanism of AANATs depends on proper orientation of the amine and thioacetyl groups, which are stabilized by three hydrogen bonds [2,15].

These inhibition experiments suggested that phenylethylamines bind to the AANAT2 catalytic pocket although they are poorly acetylated by the enzyme. This may happen if their spatial and/or electrostatic binding within the catalytic pocket does not allow efficient catalysis.

### 2.3. AANAT1a and AANAT2 Have Similar Predicted Binding Capacities

**Figure 4.**
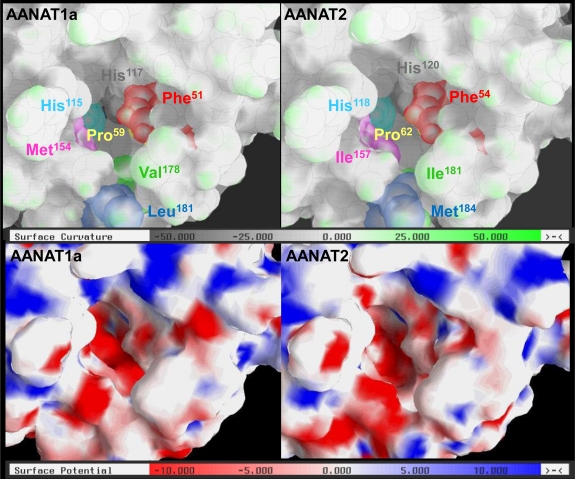
3-D surface properties of the catalytic funnel of seabream AANAT1a and AANAT2. The 3-D models of AANAT1a (left panels) and AANAT2 (right panels) were reconstructed based on the 3-D structure of the sheep AANAT complex with a bisubstrate analog [15]. The curvature analysis of the surface is shown on the upper part of the picture, with the innermost regions colored in grey and the outermost in green (see scale bar). The lower part of the picture depicts the electrostatic properties analysis of the funnel, with the positive parts colored in blue and the negative parts in red (see scale bar). Residues that form the amine binding site and catalytic residues are colored as follows: Phe^51^(AANAT1a) and Phe^54^ (AANAT2), red; Pro^59^ (AANAT1a) and Pro^62^ (AANAT2), yellow; Met^154^ (AANAT1a) and Ile^157^ (AANAT2), magenta; Val^178^ (AANAT1a) and Ile^181^ (AANAT2), green; Leu^181^ (AANAT1a) and Met^184^ (AANAT2), blue; His^115^ (AANAT1a) and His^118^ (AANAT2), cyan; His^117^ (AANAT1a) and His^120^ (AANAT2), grey. Grasp software package was used for the surface property analysis.

*Structural analysis.* Since phenylethylamines appear to bind to the catalytic pocket of AANAT2, it may be hypothesized that the kinetic differences and substrate preferences between the two enzymes stem from differences in the spatial relationships between the bound substrate and catalytic residues. This hypothesis was further explored by investigating the predicted 3-D structures of the seabream AANAT1a and AANAT2, using the structure of the sheep AANAT-bisubstrate analog complex PDB accession number: 1CJW [15] as a template. General surface and electrostatic properties were evaluated to assess the similarities and differences between the catalytic funnels of the two enzymes. Figure 4 depicts the curvature analysis of the surface (upper panels), and the electrostatic properties analysis (lower panels) of the catalytic funnel of AANAT1a and AANAT2. The overall topology of the funnel in the AANAT1a and AANAT2 models is fairly similar, with some minor differences. One stems from the presence of Leu^181^ *vs.* Met^184^ in AANAT1a and AANAT2, respectively. The longer side chain of the Met^184^ (colored blue) projects to the cavity (lower part of the pocket), and covers part of Ile^181^ of AANAT2 (colored green). It should be noted however that the Leu^181^ to Met^184^ and the Val^178^ to Ile^181^ changes have been found only in seabream AANATs, not in other known AANATs. Another topological difference, in the vicinity of the catalytic histidine residues (His^115^ and His^117^ in AANAT1a, His^118^ and His^120^ in AANAT2), is an enlargement of the cavity in AANAT2 as compared to that of AANAT1a (upper part of the cavity, Figure 4). In addition, the charge distribution between the two histidines and in the vicinity of Met^154^ (AANAT1a) and Ile^157^ (AANAT2) is different.

As suggested by the inhibition experiments, the relatively minor chemophysical differences between the two enzymes do not suggest limitations on the binding of phenylethylamines to the AANAT2 pocket. However, since the catalytic event is based on proper orientation of the ternary intermediate complex [2], these minor differences in topology and electrostatic fields may affect the efficiency of the catalysis.

### 2.4. *In Silico* Assessment of Binding Stability

Docking experiments are another bioinformatics tool that can be used to explore enzyme-substrate relationships, by predicting the probability of enzyme-substrate binding. Therefore, rigid docking experiments were performed in order to assess the binding stability of AANAT1a, AANAT2 or β5-AANAT2, and indolethylamines (serotonin and tryptamine) or phenylethylamines (dopamine and phenylethylamine), based on their geometric complementarity. Analysis of the spatial binding showed that phenylethylamines bind to the same region of AANAT2 as indolethylamines. This strengthens the hypothesis that the inhibition of tryptamine acetylation by dopamine (Figure 3) is due to competitive binding of tryptamine and dopamine within the catalytic pocket of AANAT2. Analyses of the binding stability values (supplementary data, Table S1) indicate that both AANAT1 and ANNAT2 have better geometric complementarity with indolethylamines than with phenylethylamines, as reflected in the ∼13% decrease in the binding stability values for each enzyme. This is in agreement with the measured activities of AANATs, which indicate that indolethylamines are the better substrates for both enzymes (Figure 2; [22]). Moreover, in agreement with the results of the inhibition experiments (Figure 3), stable AANAT2-phenylethylamines complexes were predicted with values that were similar (>99%) to those of AANAT1a-phenylethylamines complexes, suggesting that phenylethylamines bind AANAT1a and AANAT2 pockets with similar efficiencies. This result supports the outcome of the inhibition experiments and the predicted 3-D structures analyses, suggesting that the kinetic differences between the two enzymes stem from differences in the spatial relationships between the bound substrate and catalytic residues, which determine the proximity of the amine to the thioacetyl group and neighboring residues.

A possible extension of this hypothesis stems from the increased affinity of the AANAT2 mutants, not only to the phenylethylamines but also to the indolethylamine substrates (Table 2, Figure 2). This finding suggests that the substrate selectivity of AANAT2 is an outcome of its general low affinity, even for its preferred substrates, which results in an almost total reduction of activity with phenylethylamines. However, it should be noted that the reciprocal effect was not observed, *i.e*., the reaction rate of β5-AANAT1a with indolethylamines was only slightly decreased as compared to the wild type AANAT1a. Nevertheless, it is likely that this phenomenon at least partially contributes to the different substrate preferences of the two enzymes.

Multiple functions of fish AANATs are consistent with the hypothesis that the ancestral AANAT evolved as a detoxification enzyme in the primitive photoreceptor [30,31]. Later in evolution, when melatonin became an important signal, its increased production became a pineal gland-specific feature in order to avoid toxic processes within the visual phothoreceptor cell. Consequently, current AANAT expression in the vertebrate retina may reflect its ancestral-conserved function as a detoxification enzyme. The expression of AANAT in extra-pineal and extra-retinal sites including the brain [37–40], raises the hypothesis that this enzyme is involved in detoxification functions in these tissues. Of special interest is the idea that AANAT acetylates dopamine in the brain.

The results of the current study provide a possible molecular-structural mechanism for the substrate specificity of the two paralog AANATs, and demonstrate how changes in the coding sequence may have led to the formation of two enzymes with different biochemical properties and possibly different functions.

## 3. Experimental Section

### 3.1. Mutant Design

Candidate residues that control substrate preference of AANAT1a and AANAT2 were identified using site-directed mutagenesis. Mutations were selected based on: (1) the uniqueness of residues that characterize fish AANTA2 *vs.* fish AANAT1s and AANATs of other vertebrates (multiple sequence alignment, ClustalW 1.82); and (2) the deduced or demonstrated importance of the region for the catalytic activity or substrate binding [13,15,17]. AANTA1a and AANAT2 were mutated by reciprocal substitutions. For example, in mutation β5 (Table 1), Met^154^ in AANAT1a was changed to Ile which occupies the corresponding position in AANAT2 and *vice versa*. A description of each mutant enzyme is presented in Table 1.

### 3.2. Preparation of Wild Type and Mutant Proteins

Wild type and mutant proteins were expressed as GST fusion proteins. Seabream AANAT1a and AANAT2 expression vectors, *psb1ex* and *psb2ex*, respectively [22], were used as templates to prepare mutant protein expression vectors using the Quickchange PCR-based site-directed mutagenesis kit (Stratagene, La Jolla, CA, USA). Mutations were performed according to manufacturer’s instructions, and were verified by DNA sequencing. Plasmid vectors (pGEX-4T-1, Amersham) containing the wild type or mutant coding region were transformed into BL21 *E. coli*. Proteins were produced and purified as described [22]. Purity and concentration of the purified recombinant proteins were determined by 10% SDS-polyacrylamide gel electrophoresis and Bradford assay, respectively. Protein aliquots (10 μL) were stored at −80 °C. The preparations of the wild type and mutant AANAT2s yielded high expression and purity (∼80%) and the proteins exhibited acetylation activities (see results). Wild type AANAT1a and mutant β5-AANAT1a had lower expression, were isolated at ∼70% purity and exhibited acetylation activities. Mutant proteins α2-, β4- and αa-AANAT1a did not express well and were inactive, consistent with misfolding, and therefore were not analyzed.

### 3.3. Assay of AANAT Activity

A colorimetric assay was used to measure the activity of wild type and mutant proteins. This assay is based on the quantification of CoASH generated during acetyl transfer [12,41]. Standard reactions for wild type and mutated proteins were as previously described for AANAT1a and AANAT2 [22] in the presence of the indicated concentrations of amine substrates. Assays were done 2–3 times in duplicate. Apparent Km and Kcat values were calculated by multiple non-linear regressions, using the program GOSA [42], and are presented as mean ± SE.

A radiochemical assay [43] was used to determine the activity of wild type proteins in ‘inhibition experiments’ which were designed to determine the effect of dopamine on the acetylation of tryptamine. Wild type proteins (25 nM) were incubated with the indicated concentration of tryptamine and dopamine and 0.5 mM ^3^H-acetyl-coenzyme A (4.1 Ci/mmol, Amersham, Buckinghamshire, UK), in a final volume of 100 μL for 40 min, at optimal conditions of each enzyme. The product *N*-[^3^H]-acetyltryptamine was extracted into chloroform; this procedure does not extract acetyldopamine. The chloroform was evaporated and the residue was redissolved in scintillation fluid for counting. Assays were done twice in duplicate and are presented as mean ± SE.

### 3.4. Structural Analysis

Pairwise alignment of seabream AANAT1a (GenBank accession number: AY533402), AANAT2 (GenBank accession number: AY533403), and sheep AANAT (Uniprot accession number: 002785) (Figure 1) was done using the Needle alignment tool [44,45], and was used as a basis for the homology modeling of the 3-D models of the seabream AANAT1a and AANAT2.

The structure-modeling package JACKAL (Nest module, [46]) was used to calculate the AANAT1a and AANAT2 structures, based on the template structure of sheep AANAT in complex with a bisubstrate analog (PDB entry: 1CJW). The stereochemical quality of the models was checked using the PROCHECK suite [47]. The PROC-AVA values were 0.35 and 0.3 for AANAT1a and AANAT2, respectively, indicating high structural reliability. In addition, the resulting models were compared to models calculated using the ModBase modeling server [48]. The structure models were found to be similar in both modeling methods. Hydrogen atoms were added using H++ server [49] under the following conditions: pH = 6.5, I = 0.15 M, ɛ_in = 6, ɛ_out = 80. The 3-D calculated models of AANAT1a and AANTA2 are provided upon request. Grasp software package [50] was used for surface property analysis (same conditions as above). Assessments of the binding stability between AANAT1a, AANAT2, or β5-AANAT2 and indolethylamines (serotonin and tryptamine) or phenylethylamines (dopamine and phenylethylamine), were obtained by rigid docking based on geometric complementarity of the protein and substrate (PatchDock server, [51,52].

### 3.5. Statistical Analysis

Differences in acetylation activity between wild type AANAT1a and its mutant were evaluated using t-test. Differences between wild type AANAT2 and its mutants were evaluated using one sample t-test for phenylethylamine substrates or by analysis of variance (ANOVA) followed by Tukey’s post-hoc test for the indolethylamine substrates. To test similarity of dopamine inhibition effect between AANAT1a and AANAT2, Pearson’s correlation was carried out.

## 4. Conclusions

In the current study, the bases for the biochemical properties and substrate specificity differences between AANAT1a and AANAT2 paralogs were biochemically investigated, followed by supporting *in silico* experiments. The results suggest that the kinetics of these enzymes is determined by the accessibility of the bound substrate to catalytic residues within the catalytic pocket. This expands the understanding of substrate selectivity and mechanism of action of the AANAT enzyme family, and may be useful for the development of specific inhibitors of AANAT, which are of special interest for melatonin-related clinical applications such as sleep disorders, and jet lag or mood disorders, in which serotonin and dopamine are involved.

## Supplementary Data



## Figures and Tables

**Table 1.** Mutation sites in AANAT1a and AANAT2 and the comparable sites in the sheep AANAT.

	**Mutation sites**		

**Mutation region/name**	**AANAT1a**	**AANAT2**	**Sheep AANAT**
β5	M154I	I157M	M159
α2	-	L69R + N70H + G73T + Q74L	Q71 + H72 + T75 + L76
β4	-	S123A	A125
αa	-	M107L	L109

a Region definition is after Hickman *et al.* [15] and Dyda *et al.* [2].

b AANAT1a- α2, β4 and αa mutants were inactive and therefore were not analyzed.

**Table 2.** Apparent Kcat/Km values (mean ± SE) of wild type and mutant proteins for serotonin, tryptamine, dopamine and phenylethylamine.

	**AANAT1a**		**AANAT2**				

	**Wild type**	**β5**	**Wild type**	**β5**	**α2**	**β4**	**αa**
Serotonin	61.36 ± 4.39	64.15 ± 3.49	6.60 ± 0.54	11.34 ± 0.93	17.65 ± 1.06	18.58 ± 1.41	7.29 ± 0.54
Tryptamine	23.37 ± 1.59	14.84 ± 0.60	3.19 ± 0.25	8.50 ± 0.69	6.62 ± 0.33	9.06 ± 0.78	1.46 ± 0.003

Dopamine	7.30 ± 0.54	1.86 ± 0.49	1	2.40 ± 0.10	3.80 ± 0.07	2.48 ± 0.18	0.65 ± 0.03
Phenylethylamine	32.86 ± 2.74	5.05 ± 0.61	1	2.52 ± 0.26	4.33 ± 0.56	3.03 ± 0.09	0.61 ± 0.03

Note: Acetylation of phenylethylamine and dopamine by AANAT2 mutants are expressed relative to wild type and are indicated as shaded values.
