# Supplementary material for: Molecular Evolution of Multiple Arylalkylamine N-Acetyltransferase (AANAT) in Fish
Source: Mar Drugs. 2011 May 24;9(5):906–21. doi: 10.3390/md9050906 (PMC3111191; doi:10.3390/md9050906)
Supplement: Supplementary file 1 [file marinedrugs-09-00906-s001.pdf]

Table S1. Binding stability values of enzyme-substrate complexes.

	Complex rank	Serotonin	Tryptamine	Dopamine	Phenylethylamine
	1	3540	3466	3064	2954
AANAT1a	2	3418	3270	3030	2870
	3	3400	3222	2874	2840
	4	3364	3218	2822	2704
	5	3220	3016	2768	2632
	6	3124	2962	2580	2604
	7	2936	2896	2578	2536
	8	2920	2854	2546	2452
	9	2824	2826	2516	2388
	10	2814	2790	2492	2382
	11	2770	2732	2486	2368
	12	2762	2702	2436	2276
	13	2756	2674	2392	2268
	14	2730	2590	2368	2150
	15	2672	2578	2310	2140
	16	2618	2526	2228	2130
	17	2604	2480	2226	2116
	18	2548	2436	2188	2110
	19	2498	2410	2162	2078
	20	2494	2368		2072
	1	3506	3428	3076	2944
AANAT2	2	3394	3380	2902	2834
	3	3232	2986	2890	2716
	4	3074	2974	2780	2666
	5	3024	2910	2718	2528
	6	2994	2904	2718	2434
	7	2964	2762	2656	2396
	8	2934	2736	2634	2358
	9	2916	2700	2528	2354
	10	2754	2696	2464	2352
	11	2752	2696	2438	2326
	12	2750	2688	2430	2294
	13	2738	2652	2398	2238
	14	2730	2584	2370	2194
	15	2722	2564	2370	2150
	16	2680	2454	2366	2134
	17	2654	2450	2338	2126
	18	2648	2390	2328	2070
	19	2632	2380	2284	2062
	20	2626	2378	2240	2060

Table S1. *Cont.*

	Complex rank	Serotonin	Tryptamine	Dopamine	Phenylethylamine
	1	3500	3452	3104	2982
β5-AANAT2	2	3456	3408	3030	2930
	3	3362	2912	2958	2878
	4	2196	2910	2810	2598
	5	3188	2904	2634	2434
	6	2964	2900	2616	2374
	7	2934	2756	2560	2358
	8	2924	2722	2528	2354
	9	2754	2720	2500	2326
	10	2752	2710	2492	2292
	11	2750	2696	2464	2194
	12	2748	2688	2446	2184
	13	2746	2646	2444	2180
	14	2738	2594	2430	2152
	15	2722	2584	2370	2134
	16	2654	2522	2366	2126
	17	2632	2510	2314	2070
	18	2626	2506	2284	2062
	19	2626	2454	2246	2060
	20	2546	2448	2194	2020
